# Association between sedentary behavior and depression in US adults with chronic kidney disease: NHANES 2007–2018

**DOI:** 10.1186/s12888-023-04622-1

**Published:** 2023-03-09

**Authors:** Lin Liu, Yuqin Yan, Jingxian Qiu, Qiongmei Chen, Yujing Zhang, Yun Liu, Xiaoshi Zhong, Yan Liu, Rongshao Tan

**Affiliations:** 1 Clinical College of Medicine, Guizhou Medical University, Guiyang, China; 2 Department of Cardiology, Shenzhen Baoan Peoples Hospital, Shenzhen, China; 3 Institute of Disease-Oriented Nutritional Research, Guangzhou Red Cross Hospital of Jinan University, Guangzhou, China; 4 Department of Nephrology, Guangzhou Red Cross Hospital of Jinan University, Guangzhou, China

**Keywords:** Depression, Sedentary behavior, Physical activity, Chronic kidney disease

## Abstract

**Background:**

Depression increases the risk of adverse clinical outcomes in patients with chronic kidney disease. Physical activity has been shown to improve depressive symptoms in this population, but the relationship of sedentary behavior with depression has not been studied. In this study, we examined the relationship between sedentary behavior and depression in patients with chronic kidney disease.

**Methods:**

This cross-sectional study included 5,205 participants aged ≥ 18 years with chronic kidney disease participating in the 2007–2018 National Health and Nutrition Examination Survey. Depression was assessed using the Patient Health Questionnaire-9 (PHQ-9). Recreation activity, work activity, walking or cycling for transportation, and sedentary behavior were measured using the Global Physical Activity Questionnaire. A series of weighted logistic regression models were used to investigate the aforementioned relationship.

**Results:**

The prevalence of depression among US adults with chronic kidney disease was 10.97% in our study. In addition, sedentary behavior was strongly associated with higher levels of depressive symptoms, as measured by the PHQ-9 (P < 0.001). In the fully adjusted model, we found that compared with participants who had shorter durations of sedentary behavior, participants who had the highest durations of sedentary behavior had 1.69 times (odd ratio 1.69, 95% confidence interval: 1.27, 2.24) greater risk of being clinically depressed. After adjusting for confounding factors, subgroup analyses showed that the association between sedentary behavior and depression still existed in all stratifications.

**Conclusion:**

We found an association between longer duration of sedentary behavior and more severe depression in US adults with chronic kidney disease; however, prospective studies with larger sample sizes are still needed to confirm the effects of sedentary behavior on depression in the chronic kidney disease population.

**Supplementary Information:**

The online version contains supplementary material available at 10.1186/s12888-023-04622-1.

## Background

Depression is a common mental disorder. It is estimated that 5% of adults suffer from depression, which is also a leading cause of disability worldwide and a major contributor to the overall global burden of disease [1]. The prevalence of depression among adults aged 18 years and over in the US is approximately 8.4% (95% CI, 7.9–8.80), of which less than one-third receive antidepressant treatment [2]. The global prevalence of chronic kidney disease (CKD) is between 11 to 13%[3]. Previous studies have shown that depression is one of the most common psychiatric disorders in patients with CKD [4]. A correlational study showed a bidirectional relationship between depression and CKD, which could lead to mutual disease progression[5]. The prevalence of interview-diagnosed depression was 22.8% (confidence interval [CI], 18.6–27.6) in patients with end-stage CKD, 26.5% (95% CI, 11.1–37.2%) in CKD stages 1–5, and 25.7% (95%CI, 12.8–44.9%) in kidney transplant recipients [6]. Increasing evidence has shown that depressive symptoms are associated with an increased risk of adverse clinical outcomes in patients with CKD, including increased mortality [7] and hospitalization rates [8], and decreased quality of life [9]. Therefore, it is necessary to improve depressive symptoms in patients with CKD. However, the diagnosis and treatment of depression in patients with CKD are clinically challenging, partially because of uncertainty about the efficacy and safety of antidepressant drugs in this population [10]. Therefore, non-drug therapy is particularly important, including cognitive behavioral therapy, exercise therapy, and other methods [11].

Increasing evidence indicates that there is a relationship between physical activity (PA) levels and personal mood [12]. In addition to optimizing physical function, reducing cardiovascular risk, and improving dialysis efficacy, an exercise plan may have beneficial effects on depressive symptoms and various health-related quality-of-life indicators in CKD patients [13]. However, in patients with CKD, almost all studies have focused on exploring the relationship between PA and depression, and few studies have investigated the relationship with sedentary behavior (SB). The World Health Organization (WHO) has shown that, worldwide, one in four adults does not meet the global recommended level of PA, and the risk of death of insufficiently active people has increased by 20–30% compared with sufficiently active people[14]. A meta-analysis showed that SB is significantly associated with an increased risk of depression in the general population [15]. In addition, decreased PA levels are often observed in CKD patients, which can lead to a diminished health-related quality of life and increased morbidity and mortality[16]. Previous studies have shown that decreased estimated glomerular filtration rates (eGFR) are closely associated with prolonged durations of SB [17][18]. Currently, no study has focused on the impact of SB on depressive symptoms in patients with CKD, when taking PA into consideration. This study aimed to analyze the relationship between SB and depressive symptoms in patients with CKD.

## Methods

### Participants

The National Health and Nutrition Examination Survey (NHANES) is a nationwide survey that assesses the health and nutritional status of the non-institutionalized civilian population in the US. The study is conducted by the National Center for Health Statistics (NCHS) of the Centers for Disease Control and Prevention every two years. It has been a continuous program since 1999, using a stratified, multistage probability sampling design, with a sample of approximately 5000 people nationwide. The NHANES was approved by the NCHS Research Ethics Review Board and all participants provided written informed consent before inclusion in the study [19]. All information from the NHANES program is available and free to the public; therefore, the approval of a medical ethics committee board was not necessary.

In this cross-sectional study, we used data from 6 NHANES cycles: 2007–2008, 2009–2010, 2011–2012, 2013–2014, 2015–2016, and 2017–2018. The following inclusion criteria were applied: (1) being interviewed and examined; (2) age ≥ 18 years; (3) diagnosed with CKD; in the NHANES database, detail four situations in the questionnaire on SB and Depression: Know, Refused, Don’t Know, and Missing. Exclusion criteria were applied to participants who lacked information concerning SB and Depression.

### Assessment of CKD

The Modification of Diet in Renal Disease Study Equation was used in this study to calculate the eGFR [20]. CKD was diagnosed with an eGFR < 60 ml/min per 1.73 m^2^ or urine albumin-to-creatinine ratio (UACR) ≥ 30 mg/g[21]. In this study, CKD was defined as G1-G3a in the early stage and G3b-G5 in the late stage according to the GFR stage, excluding hemodialysis patients [22].

### Assessment of depression

Participants’ depression was evaluated in a mobile examination center by trained interviewers using a computer-assisted personal interviewing system. The Patient Health Questionnaire (PHQ-9) score was used to assess depression. An individual participant data meta-analysis compared the PHQ-9 score with the diagnosis of major depression in a validated diagnostic interview. It was found that PHQ-9 was more sensitive than the semi-structured diagnostic interview (designed for the management of clinicians). A cut-off score of 10 or above maximized the overall sensitivity and specificity of the whole and subgroups[23]. Additionally, another article found that when the interview results of mental health professionals were used to evaluate the effectiveness of PHQ-9 results, the sensitivity and specificity of PHQ-9 score ≥ 10 for major depression could reach as high as 88%[24]. The PHQ-9 is a nine-item depression screening instrument that evaluates the frequency of symptoms of depression in the past two weeks [24]. Response categories of “not at all,” “several days,” “more than half the days,” and “nearly every day” were scored 0, 1, 2 and 3 respectively. Summary scores ranged from 0 to 27. Depression was defined using a cutoff score of 10 or higher, a well-validated cutoff point used in primary care settings[24].

### Assessment of PA and SB

As independent variables, information on PA and SB was self-reported in NHANES using the Global Physical Activity Questionnaire (GPAQ). The GPAQ has been validated in other populations, with the reliability of moderate to substantial strength (Kappa0.67 to 0.73; Spearman’s rho 0.67 to 0.81), and concurrent validity between International Physical Activity Questionnaire (IPAQ) and GPAQ is moderate to strongly positive (range 0.45 to 0.65). In short, GPAQ provides repeatable data and shows a moderately strong positive correlation with IPA[25]. According to the WHO Guidelines on PA and SB[26], participants who engaged in ≥ 150 min/week of moderate-intensity aerobic PA, ≥ 75 min/week of vigorous-intensity aerobic PA, or had an equivalent combination of moderate and vigorous PA (1 min of vigorous PA is equivalent to 2 min of moderate PA) totaling at least 150 min/week were defined as meeting the guidelines. According to the reported number of days and time in minutes spent on moderate or vigorous work activity and moderate or vigorous recreational activity, participants were classified as having insufficient moderate-to-vigorous work activity (MVWA) (˂150 min/week), insufficient moderate-to-vigorous recreational activity (MVRA) (˂150 min/week), sufficient MVWA (≥ 150 min/week), and sufficient MVRA (≥ 150 min/week). In addition, based on the self-reported number of days and time spent walking/cycling, sufficient walking/cycling was defined as walking/bicycling for at least 150 min per week. Participants whose walking/cycling time was less than 150 min/week were defined as having insufficient walking/cycling. SB is defined as activities that do not increase energy expenditure above the resting level (i.e., < 1.5 metabolic equivalents) and includes time spent on activities such as sitting and lying down during waking hours, working on a computer, watching TV, and engaging in other forms of screen-based entertainment[27]. The duration of SB was calculated using the self-reported time usually spent sitting on a typical day (PAD 680), ranged from 0 to 1320 min per day [28].

### Assessment of covariates

Demographic and sociodemographic factors included age, sex (men/women), race (Mexican American, non-Hispanic white, non-Hispanic black, and others), education (did not finish high school, finished high school, and some college or above), marital status (never married, divorced/separated/widowed, married, or living with a partner), employment status, and income. Information on employment status was obtained from an occupation questionnaire, and job status was classified into two groups (yes or no). Socioeconomic status was classified using the ratio of family income to poverty (poverty index ratio [PIR]), with participants categorized as poor (PIR ≤ 1.3), near poor (1.3 < PIR < 3.5), and non-poor (PIR ≥ 3.5) [29]. Lifestyle-related behaviors included smoking status (never smoked, current smokers) and alcohol consumption status (never, former drinker, light drinker, moderate drinker, and heavy drinker)[30]. Body mass index (BMI) was calculated as the measured weight (kg) divided by the square of height (m^2^). Preexisting comorbidities included hypertension (defined as a history of physician-diagnosed hypertension, with a measured average systolic blood pressure of ≥ 140 mmHg, a measured average diastolic blood pressure of ≥ 90 mmHg, or reported use of antihypertensive agents [31] and diabetes (defined as a history of physician-diagnosed diabetes, with a fasting plasma glucose ≥ 7.0 mmol/L, glycosylated hemoglobin [HbA1c] ≥ 6.5%, or the use of antihyperglycemic agents) [32]. According to the 2012 clinical practice guidelines for global results of chronic kidney disease improvement, CKD should be diagnosed according to etiology, GFR classification, and proteinuria classification[22].

### Statistical analysis

All statistical analyses were performed using R (http://www.R-project.org, Version 4.2.1 with package “forestplot”) and Empower Stats (http://www.empowerstats.com) software. In the calculation for all estimates, we used 11-year sample weights following the analytical guideline edited by the NCHS to ensure that the NHANES data would represent the civilian non-institutionalized US population. Patients were divided into groups with and without depression. Categorical variables were expressed as frequencies (N) and percentages (%). Continuous variables with a normal distribution were presented as mean ± standard deviation (SD). Non-normal continuous variables were reported as medians with interquartile ranges. A weighted chi-square test and weighted linear regression model were used to test the differences between the groups. Because the durations of SB and PA were not normally distributed, we performed a natural log transformation. Multiple imputations by chained equations were used to deal with missing covariate data [33], and sensitivity analysis was further applied (Supplementary material 1). Multivariate binary logistic regression analyses were performed to confirm the association between SB and depression in patients with CKD after adjustment for confounding factors. Additionally, we performed subgroup analyses.

In the current study, age, sex, race, education, marital status, PIR, smoking status, alcohol consumption status, employment status, BMI (kg/m^2^), eGFR (ml/min), UACR (mg/g), hypertension, diabetes, duration of walking or cycling for transportation (min/week), duration of work activity (min/week), and duration of recreational activity (min/week) were considered as potential confounders and adjusted. Statistical significance was defined as a two-sided P-value of < 0.05.

## Results

### Study population and clinical characteristics

**Fig. 1:**
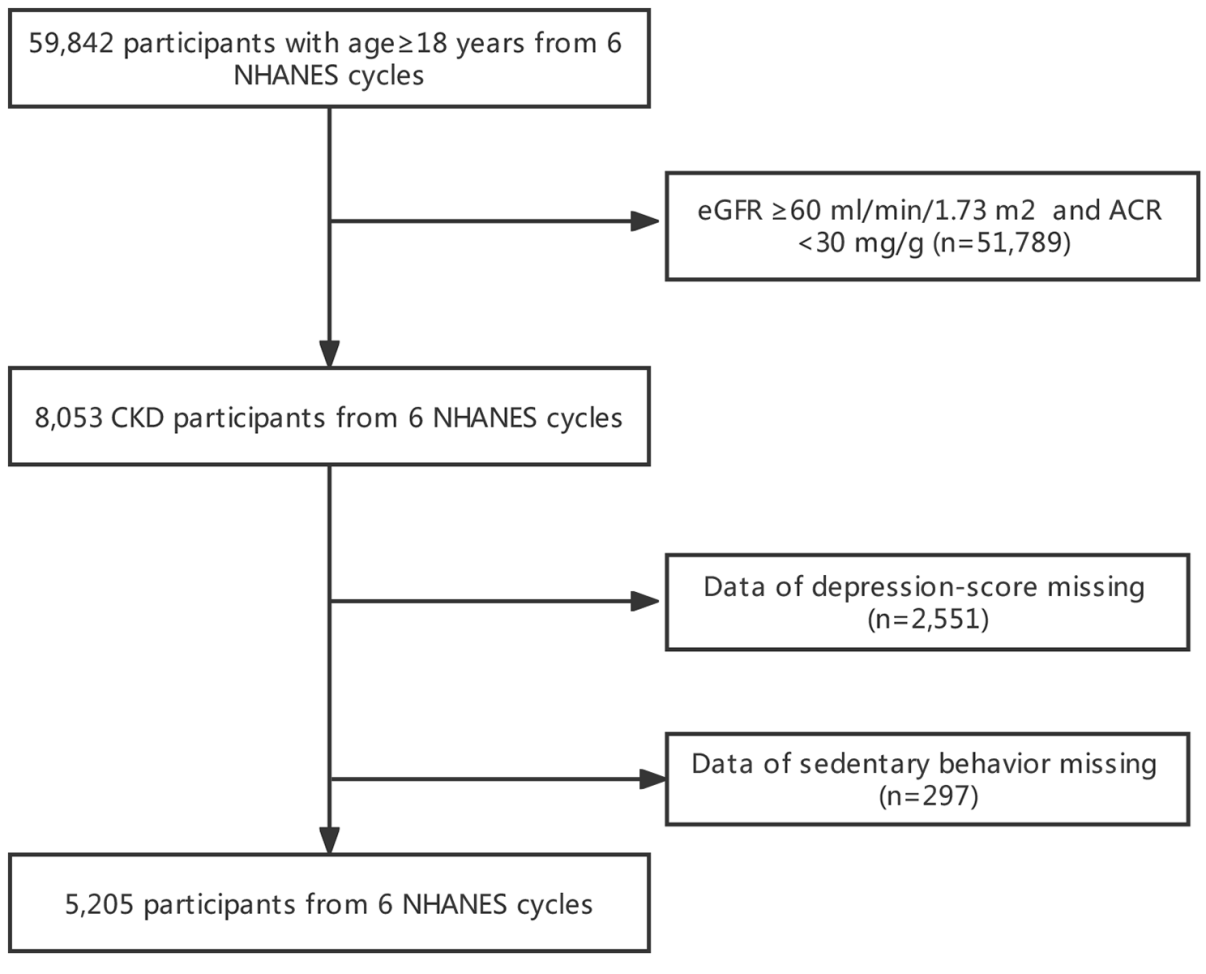
Sample selection flowchart from National Health and Nutrition Examination Survey (NHANES2007-2018).

A total of 5,205 participants (male: female 2,500:2,705) were included in the study, with a mean age of 62.23 ± 17.34 years, of whom 571 (10.97%) had suffered from depression defined by a PHQ-9 score ≥ 10. A flowchart of the sample selection process is shown in Fig. 1. Participants with depression were more likely to be younger, female, widowed, divorced, or separated, or have a higher education level, higher BMI, higher levels of UACR, higher prevalence of hypertension or diabetes, higher consumption of alcohol or cigarettes, lower PIR, longer durations of SB, or shorter durations of recreational activities (all P < 0.05) (Table 1). The participants were also divided into three groups according to tertiles of SB duration. Compared with participants with shorter durations of SB (90-270 min/d), participants with the longest duration of SB (480–1320 min/d) were older, were more likely to be non-Hispanic white and other ethnic groups, and married or living with their partners. They also had higher levels of education and PIR, higher smoking, higher BMI, higher prevalence of hypertension and diabetes, higher depression scores, and lower eGFR (all P < 0.05) (Table 2).

**Table 1:** Characteristics of the study participants stratified by depression conditions (weighted)

Variables	Patients without depression (n = 4634)	Patients with depression (n = 571)	P-value
**Age (years)**	60.46 (59.66, 61.26)	58.01 (56.30, 59.71)	0.0121
**Gender, n (%)**			0.0012
Female	2356 (55.77)	349 (64.52)	
Male	2278 (44.23)	222 (35.48)	
**Race, n (%)**			0.1185
Mexican American	330 (4.60)	59 (6.42)	
Non-Hispanic white	1357 (49.66)	163 (45.14)	
Non-Hispanic black	749 (8.53)	93 (10.27)	
Other races	2198 (37.21)	256 (38.17)	
**Marital status, n (%)**			< 0.0001
Never married	528 (11.36)	78 (11.92)	
Married or living with a partner	2576 (59.44)	244 (46.51)	
Widowed, divorced, or separated	1530 (29.20)	249 (41.57)	
**Education, n (%)**			< 0.0001
Under high school graduate	1271 (18.82)	230 (29.95)	
High school graduate and above	3363 (81.18)	341 (70.05)	
**Current Smoke, n (%)**	2253 (47.72)	343 (62.90)	< 0.0001
**Alcohol consumption status, n (%)**			0.0186
Never	1169 (27.65)	125 (22.47)	
Former	1601 (34.53)	187 (36.14)	
Mild	895 (18.58)	119 (17.32)	
Moderate	510 (10.45)	63 (10.92)	
Heavy	459 (8.79)	77 (13.14)	
**Employment status**			0.7284
Yes	3175 (69.97)	398 (70.81)	
No	1459 (30.03)	173 (29.19)	
**Poverty index ratio**	2.78 (2.69, 2.86)	1.89 (1.70, 2.08)	< 0.0001
**BMI (Kg/m** ^**2**^ **)**	30.00 (29.65, 30.36)	32.51 (31.44, 33.58)	0.0001
**eGFR (ml/min)**	72.52 (71.41, 73.64)	74.71 (71.85, 77.56)	0.1552
**Urinary albumin/creatinine ratio (mg/g)**	175.67 (155.45, 195.90)	267.98 (203.45, 332.51)	0.0073
**Hypertension, n (%)**	3320 (66.90)	433 (74.64)	0.0066
**Diabetes, n (%)**	1832 (33.89)	297 (46.74)	< 0.0001
**Walk or cycle for transportation (min/w)**	54.19 (46.54, 61.83)	40.71 (27.93, 53.50)	0.0706
**work activity (min/w)**	405.72 (365.66, 445.79)	388.32 (294.07, 482.56)	0.7183
**recreational activity (min/w)**	143.66 (128.10, 159.22)	60.35 (43.90, 76.80)	< 0.0001
**Sedentary behavior (min/d)**	394.40 (385.68, 403.12)	435.44 (412.71, 458.17)	0.0012
**Stage of CKD, n (%)**			0.7143
Early stage	3851 (85.74)	469 (85.03)	
Advanced stage	783 (14.26)	102 (14.97)	
**Stage of UACR**			< 0.0001
A1	1394 (32.64)	120 (21.32)	
A2	2675 (57.16)	343 (61.09)	
A3	565 (10.21)	108 (17.59)	

Note: CKD, chronic kidney disease; Ref, reference; UACR, urinary albumin/creatinine ratio (mg/g). For continuous variables: survey-weighted mean (95% CI), P-value was determined by survey-weighted linear regression (svyglm); for categorical variables, (N-observe, N-represent) survey-weighted percentage (95% CI), P-value was determined using a survey-weighted chi-square test (suitable)

**Table 2:** Characteristics of the study participants stratified by duration of sedentary behavior (weighted)

Variables	Low (90-270 min/d) (n = 1718)	Middle (300-420 min/d) (n = 1540)	High (480-1320 min/d) (n = 1947)	P-value
**Age (years)**	58.44 (57.19, 59.69)	60.27 (59.08, 61.45)	61.57 (60.68, 62.46)	0.0002
**Gender, n (%)**				0.6733
Female	885 (55.62)	805 (57.79)	1015 (56.45)	
Male	833 (44.38)	735 (42.21)	932 (43.55)	
**Race, n (%)**				< 0.0001
Non-Hispanic white	351 (38.66)	474 (50.72)	695 (56.28)	
Non-Hispanic black	233 (8.09)	252 (8.41)	357 (9.38)	
Mexican American	178 (7.20)	102 (4.17)	109 (3.36)	
Other races	956 (46.05)	712 (36.70)	786 (30.99)	
**Marital status, n (%)**				0.0071
Never married	181 (10.57)	192 (13.57)	233 (10.42)	
Married or living with a partner	995 (62.30)	828 (56.82)	997 (56.11)	
Widowed, divorced, or separated	542 (27.13)	520 (29.61)	717 (33.47)	
**Education, n (%)**				0.0016
Under high school graduate	567 (22.94)	427 (19.25)	507 (17.99)	
High school graduate and above	1151 (77.06)	1113, (80.75)	1440 (82.01)	
**Employment status**				0.2070
Yes	1205 (72.19)	1027 (68.75)	1341 (69.39)	
No	513 (27.81)	513 (31.25)	606 (30.61)	
**Poverty index ratio**	2.53 (2.42, 2.65)	2.62 (2.52, 2.73)	2.87 (2.74, 3.00)	0.0001
**Current Smoke, n (%)**	800 (47.11)	782 (47.43)	1014 (52.08)	0.0431
**Alcohol consumption status, n (%)**				0.2489
Never	417 (26.70)	372 (25.48)	505 (28.80)	
Former	599 (34.67)	530 (35.41)	659 (34.14)	
Mild	334 (119.65)	329 (20.11)	351 (16.29)	
Moderate	192 (9.58)	149 (9.70)	232 (11.80)	
Heavy	176 (9.40)	160 (9.30)	200 (8.97)	
**Walk or cycle for transportation (min/w)**	0.40 (0.34, 0.46)	0.41 (0.36, 0.47)	0.41 (0.36, 0.46)	0.9553
**work activity (min/w)**	0.81 (0.74, 0.88)	0.85 (0.77, 0.94)	0.91 (0.83, 1.00)	0.2905
**recreational activity (min/w)**	0.76 (0.69, 0.83)	0.74 (0.68, 0.81)	0.79 (0.72, 0.87)	0.6166
**BMI (Kg/m** ^**2**^ **)**	29.38 (28.87, 29.88)	29.66 (29.18, 30.13)	31.36 (30.82, 31.90)	< 0.0001
**Hypertension, n (%)**	1185 (64.46)	1144 (67.92)	1424 (69.86)	0.0266
**Diabetes, n (%)**	683 (31.61)	604 (32.08)	842 (40.12)	< 0.0001
**eGFR (ml/min)**	77.22 (75.23, 79.21)	72.25 (70.54, 73.96)	69.62 (67.94, 71.31)	< 0.0001
**Urinary albumin/creatinine ratio (mg/g)**	168.57 (137.32, 199.83)	176.05 (138.53, 213.57)	203.11 (172.86, 233.35)	0.2838
**Stage of CKD, n (%)**				< 0.0001
Early stage	1500 (89.41)	1273 (86.22)	1547 (82.35)	
Advanced stage	218 (10.59)	267 (13.78)	400 (17.65)	
**Stage of UACR**				0.0678
A1	461 (29.54)	491 (33.72)	562 (31.48)	
A2	1052 (59.91)	864 (57.20)	1102 (55.95)	
A3	205 (10.55)	185 (9.09)	283 (12.57)	
**Depression score**	2.90 (2.67, 3.13)	3.47 (3.13,3.81)	3.86 (3.58,4.14)	< 0.0001

Note: CKD, chronic kidney disease; Ref, reference; UCAR, urinary albumin/creatinine ratio (mg/g). For continuous variables: survey-weighted mean (95% CI), P-value was determined by survey-weighted linear regression (svyglm); for categorical variables, (N-observe, N-represent) survey-weighted percentage (95% CI), P-value was determined using a survey-weighted chi-square test (suitable)

### Association between duration of SB and depression

Three multivariate binary logistic regression models were constructed (weighted). The first was unadjusted, the second was partially adjusted (adjusted for age, sex, race, education, marital status, PIR, smoking status, alcohol consumption status, and employment status), and the third was fully adjusted (model 2 adjusted for BMI (kg/m^2^), eGFR (ml/min, ACR [mg/g]), hypertension, diabetes, walking or cycling for transportation (min/week), work activity (min/week), and recreational activity (min/week)). The fully adjusted model demonstrated an inverse relationship between SB and depression (adjusted odds ratio [OR] = 2.53, 95% CI = 1.49–4.30). That is to say, depression patients are more prone to sedentary behavior, and 2.53 times more than non-depression patients. We also observed that the group with the longest duration of SB was 1.69 (95% CI: 1.27, 2.24) times more likely to develop depression than the group with the shortest duration of SB in model 3 (Table 3).

**Table 3:** **Associations between Sedentary behavior and Depression by binary logistic regression models (weighted)**

Variable	No. of subjects	Depression score ≥ 10 score		Results from logistic regression analysis		
		No. of cases	%	Model 1 OR, 95% CI	Model 2 OR, 95% CI	Model 3 OR, 95% CI
Sedentary behavior (min/day)	5205	571	10.97	2.30 (1.32, 4.01)	3.12(1.83,5.34)	2.53(1.49,4.30)
**Sedentary behavior sub-group (min/day)**						
90–270	1718	158	7.55	Ref	Ref	Ref
300–420	1540	157	8.57	1.15 (0.88, 1.51)	1.24 (0.92, 1.66)	1.21(0.90, 1.61)
480–1320	1947	256	11.70	1.62 (1.24, 2.12)	1.88 (1.42, 2.47)	1.69(1.27, 2.24)

Note: Ref, reference. OR, Odds ratio. 95% CI, 95% confidence interval. Data in model 1 were unadjusted; Model 2 was adjusted for age, sex, race, education, marital status, poverty index ratio, smoking status, alcohol consumption status, and employment status; Model 3 was further adjusted for BMI (Kg/m^2^), eGFR (ml/min), UACR (mg/g), hypertension, diabetes, walking or cycling for transportation (min/week), work activity(min/week), and recreational activity(min/week) on the basis of Model 2

### Subgroup analyses

**Fig. 2:**
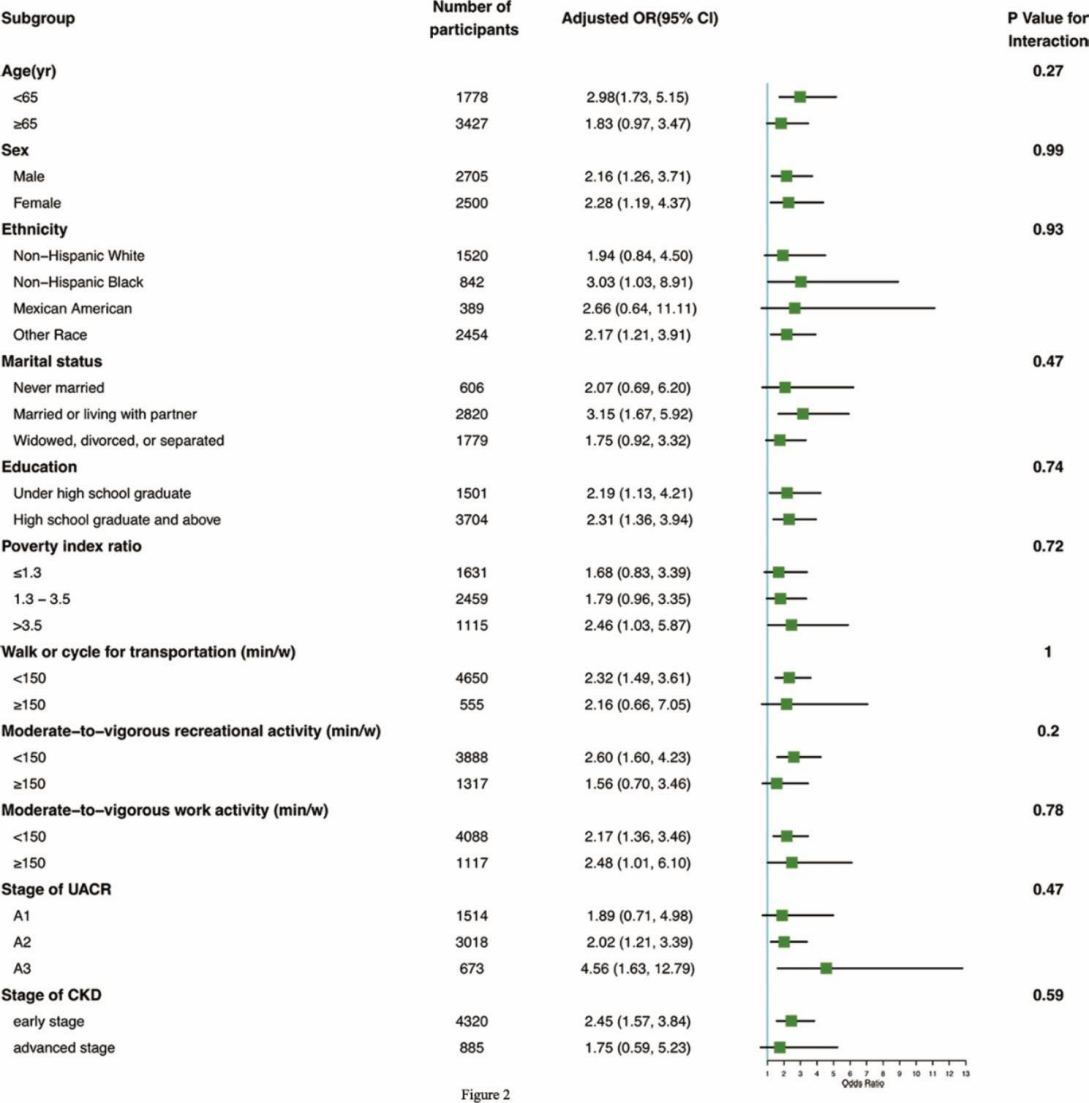
The association between sedentary behavior and depression by stratified analyses Associations between sedentary behavior and depression stratified by the basic characteristics of the population, income level, physical activity, and progression of CKD. Model adjusted for age, gender, race, education, marital status, poverty index ratio, smoking status, alcohol consumption status, employment status, BMI (Kg/m^2^) eGFR(ml/min), UACR (mg/g), hypertension, diabetes, walking or cycling for transportation (min/week), work activity (min/week), recreational activity (min/week)

After stratification by age (≥ 65 years or < 65 years), sex, race, education, marital status, PIR, walking or cycling for transportation (min/week), work activity (min/week), recreational activity (min/week), stage of CKD, and urinary albumin/creatinine ratio, subgroup analyses showed that an inverse relationship still existed between SB and depression in all stratifications (Fig. 2). However, no interaction was detected in any subgroup.

## Discussion

In this cross-sectional study of more than 5000 patients with CKD, SB was associated with depression, as measured by the PHQ-9, a widely used and well-validated psychometric instrument[23, 24]. Meanwhile, in each subgroup analysis, this relationship remained independent. In addition, after adjustment for prespecified confounders, participants in the longest duration group of SB (480-1320 min/d) had a 1.69 times greater risk of clinical depression defined by a cutoff of > 10 using the PHQ-9 than those who had the shortest duration of SB (90-270 min/d). This finding is consistent with the results of previous studies in the general population [15].

Our study shows that the prevalence of depression in patients with CKD aged 18 years and above in the US is 10.97%, which is significantly higher than the prevalence of depression in the general population of the US [1] but lower than the global prevalence of depression in patients with CKD [6]. The potential cause of this phenomenon may be different screening tools for depression, but it still highlights the importance of depression as a comorbidity in the population with CKD.

In this study, we followed the abovementioned definition of SB[27]. Many observational studies have shown that prolonged SB, independent of moderate PA, is associated with an increased risk of adverse health outcomes in the general population [34]. Some studies have also shown that in adults with other chronic diseases such as type 2 diabetes [35] and acute coronary syndrome [36], there were associations between longer durations of SB and depression.

Therefore, it is important to strengthen PA and change cognitive-behavioral patterns to reduce SB to improve depression. A systematic evaluation and meta-analysis of 191,130 participants showed that even if the PA level is lower than the recommended level for public health, it has significant benefits for depressive symptoms [37].

However, the CKD population is exposed to a variety of factors that predispose to decreased PA levels, including the disease itself and its complications, such as protein energy consumption, muscle loss, anemia, vascular dysfunction, and neuropathy [38, 39]. In addition, in an observational study of a CKD population, it was found that the strong association between mental health disorder status and SB could not be explained by demographics, smoking, comorbidities, nutrition, and inflammatory factors[40]. Therefore, compared to the general population, individuals with CKD are more inclined to have a sedentary lifestyle. Previous studies have shown that the progression of CKD and decrease in eGFR are closely related to prolonged sedentary time [18]. When our study was grouped by tertiles of sedentary duration, it was also found that the higher SB group had lower eGFR and higher sedentary behavior in the early or late stages of kidney disease (P < 0.0001). Increased PA has been associated with improved renal function [41], improved quality of life [42], and reduced all-cause mortality [43] in patients with CKD. Therefore, improving the maladaptive patterns of thinking and behavior associated with sedentary habits and strengthening PA can help patients with CKD make meaningful lifestyle changes, thereby improving their clinical outcomes.

Previous studies have shown that an increase in SB leads to the progression of CKD and depression[15][17], and CKD and depression may form a positive feedback loop[5]; however, the pathophysiological mechanisms are still unclear. Several previous randomized controlled trial studies have shown that in individuals with a sedentary lifestyle, autonomic and inflammatory responses to stress may be exacerbated, increasing the independent deleterious effects of SB on mood, especially anxiety symptoms[44], possibly through inflammatory changes [45]. Other studies have also shown that SB is associated with inflammatory status, which is represented by C-reactive protein [45] and interleukin 6[44]. Although these studies have not provided any direct causal evidence, patients with CKD often have a persistent inflammatory status[46], which may be the common pathway of the vicious cycle among the three. In addition, evidence has shown that both depression [47] and SB[48] are associated with cognitive impairment. Compared to the general population, patients with CKD have a much higher risk of cognitive impairment. Decreased eGFR and albuminuria are both related to the development of cognitive impairment and poor cognitive function [49] cognitive impairment may also play a role in the link between SB and depression. The American College of Sports Medicine recently provided a framework to encourage patients to sit less, move more, and use exercise as a therapy for treating chronic diseases. Reducing SB is considered the basis of any exercise prescription for patients of all ages and with chronic diseases. Evidence supporting the encouragement of exercise comes from observational studies, which showed that walking more steps per day is negatively related to the risk of mortality and is related to walking intensity (gait frequency) [50]. Similar conclusions may be applied to patients with CKD.

The strengths of the current study include the use of a large sample of data from a nationally representative sample of US adults and adjustment for confounding factors in different multivariate binary logistic regression models. However, the study inevitably had limitations. First, it was an observational cross-sectional study; therefore, causal conclusions cannot be drawn. Second, some of the data from the questionnaires, such as history of alcohol consumption, smoking, chronic diseases, time spent in sedentary behavior and physical activity, may have been subject to recall bias resulting in inaccurate results. Besides, there may be other potential confounding factors that have not been adjusted, such as the cognitive level of participants. Finally, although the PHQ-9 can be used to screen for depression, further diagnosis must adhere to the guidelines of the Diagnostic and Statistical Manual of Mental Disorders [51].

In conclusion, we observed that a longer duration of SB was positively associated with depressive symptoms in patients with CKD; however, prospective studies with larger sample sizes are needed to confirm the effects of SB on depression in the CKD population.

## Electronic supplementary material

Below is the link to the electronic supplementary material.

Supplementary Material 1: Sensitivity Analyses for associations between Sedentary behavior and Depression by binary logistic regression (weighted)

## Data Availability

The datasets analyzed during the current study are publicly available for download from the National Center for Health Statistics at the Centers for Disease Control. https://www.cdc.gov/nchs/nhanes/index.htm.

## List of Abbreviations

CKD: Chronic kidney disease
PA: Physical activity
SB: Sedentary behavior.
PHQ-9: Patient Health Questionnaire-9.
eGFR: Estimated glomerular filtration rate.
NHANES: National Health and Nutrition Examination Survey.
GPAQ: Global Physical Activity Questionnaire.
UACR: Urine albumin-to-creatinine ratio.
PIR: Poverty index ratio.
BMI: Body-mass index.
