# Correction to: Association between sedentary behavior and depression in US adults with chronic kidney disease: NHANES 2007–2018

**DOI:** 10.1186/s12888-023-04707-x

**Published:** 2023-04-13

**Authors:** Lin Liu, Yuqin Yan, Jingxian Qiu, Qiongmei Chen, Yujing Zhang, Yun Liu, Xiaoshi Zhong, Yan Liu, Rongshao Tan

**Affiliations:** 1 Clinical College of Medicine, Guizhou Medical University, Guiyang, China; 2 Department of Cardiology, Shenzhen Baoan Peoples Hospital, Shenzhen, China; 3 Institute of Disease-Oriented Nutritional Research, Guangzhou Red Cross Hospital of Jinan University, Guangzhou, China; 4 Department of Nephrology, Guangzhou Red Cross Hospital of Jinan University, Guangzhou, China

Following publication of the original article [1], the authors identified that Yun Liu was omitted as co-corresponding author.

The author group has been updated above and the original article [1] has been corrected.

## References

[1] Liu, BMC Psychiatry. (2023) Association between sedentary behavior and depression in US adults with chronic kidney disease: NHANES 2007–2018 (2023) 23:148 DOI: 10.1186/s12888-023-04622-1 10.1186/s12888-023-04622-1 PMC9996893 36894924

