# Supplementary material for: Association between sedentary behavior and depression in US adults with chronic kidney disease: NHANES 2007–2018
Source: BMC Psychiatry. 2023 Mar 9;23:148. doi: 10.1186/s12888-023-04622-1 (PMC9996893; doi:10.1186/s12888-023-04622-1)
Supplement: Supplementary file 1 — Supplementary Material 1: Sensitivity Analyses for associations between Sedentary behavior and Depression by binary logistic regression (weighted) [file 12888_2023_4622_MOESM1_ESM.docx]

**Supplementary Material 1. Sensitivity Analyses for associations between Sedentary behavior and Depression** **by binary logistic regression (weighted).**

| **Variable** | **No. of subjects** | **Depression**  **Results from logistic regression analysis** | | | | | |
| --- | --- | --- | --- | --- | --- | --- | --- |
|  |  | **Raw data**  **OR, 95% CI** | **Group 1**  **OR, 95% CI** | **Group 2**  **OR, 95% CI** | **Group 3**  **OR, 95% CI** | **Group 4**  **OR, 95% CI** | **Group 5**  **OR, 95% CI** |
| **Sedentary behavior (min/day)** | 5205 | 2.29 (0.99, 5.30) | 2.54 (1.43, 4.49) | 2.44 (1.37, 4.36) | 2.52 (1.41, 4.50) | 2.49 (1.41, 4.41) | 2.57 (1.44, 4.61) |
| **Sedentary behavior sub-group (min/day)** |  |  |  |  |  |  |  |
| **90-270** | 1718 | Ref. | Ref. | Ref. | Ref. | Ref. | Ref. |
| **300-420** | 1540 | 1.11 (0.70, 1.77) | 1.25 (0.92, 1.70) | 1.24 (0.91, 1.69) | 1.25 (0.91, 1.70) | 1.23 (0.90, 1.68) | 1.25 (0.92, 1.70) |
| **480-1320** | 1947 | 1.65 (1.07, 2.52) | 1.71 (1.27, 2.30) | 1.71 (1.26, 2.31) | 1.72 (1.27, 2.32) | 1.69 (1.26, 2.27) | 1.72 (1.27, 2.32) |

Note: Ref, reference. OR, Odds ratio. 95% CI, 95% confidence interval**.** Data in the model was adjusted for age, sex, race, education, marital status, poverty index ratio, smoking status, alcohol consumption status, employment status, BMI (Kg/m^2^), eGFR (ml/min), UACR (mg/g), hypertension, diabetes, walking or cycling for transportation(min/week), work activity(min/week), and recreational activity(min/week).
